# *In Vitro* Analysis of Metabolites Secreted during Infection of Lung Epithelial Cells by *Cryptococcus neoformans*

**DOI:** 10.1371/journal.pone.0153356

**Published:** 2016-04-07

**Authors:** Kah Leong Liew, Jap Meng Jee, Ivan Yap, Phelim Voon Chen Yong

**Affiliations:** 1 School of Biosciences, Taylor’s University, Subang Jaya, Selangor Darul Ehsan, Malaysia; 2 Department of Life Sciences, International Medical University, Kuala Lumpur, Malaysia; University of Michigan Health System, UNITED STATES

## Abstract

*Cryptococcus neoformans* is an encapsulated basidiomycetous yeast commonly associated with pigeon droppings and soil. The opportunistic pathogen infects humans through the respiratory system and the metabolic implications of *C*. *neoformans* infection have yet to be explored. Studying the metabolic profile associated with the infection could lead to the identification of important metabolites associated with pulmonary infection. Therefore, the aim of the study was to simulate cryptococcal infection at the primary site of infection, the lungs, and to identify the metabolic profile and important metabolites associated with the infection at low and high multiplicity of infections (MOI). The culture supernatant of lung epithelial cells infected with *C*. *neoformans* at MOI of 10 and 100 over a period of 18 hours were analysed using gas chromatography mass spectrometry. The metabolic profiles obtained were further analysed using multivariate analysis and the pathway analysis tool, MetaboAnalyst 2.0. Based on the results from the multivariate analyses, ten metabolites were selected as the discriminatory metabolites that were important in both the infection conditions. The pathways affected during early *C*. *neoformans* infection of lung epithelial cells were mainly the central carbon metabolism and biosynthesis of amino acids. Infection at a higher MOI led to a perturbance in the β-alanine metabolism and an increase in the secretion of pantothenic acid into the growth media. Pantothenic acid production during yeast infection has not been documented and the β-alanine metabolism as well as the pantothenate and CoA biosynthesis pathways may represent underlying metabolic pathways associated with disease progression. Our study suggested that β-alanine metabolism and the pantothenate and CoA biosynthesis pathways might be the important pathways associated with cryptococcal infection.

## Introduction

Cryptococcosis is a systemic fungal infection most commonly attributed to infection by *Cryptococcus neoformans* [1]. Inhalation of *C*. *neoformans* by immunocompetent individuals would lead to minimal symptoms, if any, but individuals with reduced immunological functions would have pulmonary infections and subsequently suffer from meningoencephalitis [2]. The Center for Disease Control and Prevention (USA) estimated one million cases of cryptococcal meningitis annually, with a staggering death toll of 625,000 [3,4]. Sub-Saharan Africa and Southeast Asia, a region where over 80% of the world’s HIV-affected population lives, bears the greatest burden of this disease, with mortality rates of 50% to 70% [1, 3]. As cryptococcosis is a non-notifiable disease, the incidence counts and the mortality rates might be underestimated [5].

Metabolic response has been used to reflect the changes present in biological functions in response to genetic or environmental changes [6]. The metabolic adaptation in *C*. *neoformans* during early murine pulmonary infection has been documented and several genes were found to have a role in its virulence [7]. A study on the effects of iron availability toward the host-pathogen interaction in *C*. *neoformans* has also been reported [8]. Metabolomics involve the quantitative measurement of metabolic responses of multicellular systems to pathophysiological stimuli [9]. Metabolic biomarkers could be either a single molecule or a pattern of several molecules that could potentially predict disease, measure progression and even monitor therapy. Metabolic profiling has been associated with the study of potential biomarkers in several diseases such as coronary heart disease and hypertension, liver and epithelial ovarian cancer, type 2 diabetes, motor neuron disease, myocardial ischaemia, Huntington’s disease and schizophrenia [10].

The clinical manifestations of *C*. *neoformans* infection varies from being asymptomatic or cough to fever, pneumonia, meningoencephalopathy, dissemination to multiple sites of the body and even death [2]. The traditional way of diagnosis for cryptococcosis involves the successful culture or demonstration of encapsulated yeasts in India ink preparations from cerebrospinal fluid but the method is cumbersome and time consuming [11]. Serological methods such as latex agglutination test (LAT) as a complementary procedure to support diagnosis has been implemented but there are limitations of false positivity and negativity as well as the difficulty of its interpretation in borderline cases [11]. To date, studies on the possible biomarkers associated with *C*. *neoformans* infection of the lung have not been documented. Mice infected with *C*. *neoformans* exhibited an increased allergic response and a more severe pulmonary infection associated with an increase in fungal load [12], but the effects of fungal infection load on the metabolic response have yet to be established. Therefore, in this study, we examined the metabolic profiles of *C*. *neoformans* infection of lung epithelial cells at low and high multiplicity of infection (MOI) as well as the important metabolites associated with the infection.

## Materials and Methods

### Chemicals and reagents

DL-Alanine-2, 3, 3, 3-d_4_, N-methyl-N-(trimethylsilyl) trifluoroacetamide (MSTFA), pyridine and methoxyamine hydrochloride were purchased from Sigma-Aldrich.

### Yeast strain and culture conditions

*Cryptoccoccus neoformans* var. *grubii* strain H99 (ATCC 208821) was used in this study. All *C*. *neoformans* cultures were cultured and maintained in Sabouraud dextrose agar and broth (Becton Dickinson, USA) at 37°C. For experimental work, approximately 10^5^ cells/ml of *C*. *neoformans* were grown for 28 hours at 37°C in Sabouraud dextrose broth until the mid-log phase. The yeast cells were obtained by centrifugation and washed twice in phosphate-buffered saline (PBS). The cell number was estimated by counting the number of yeasts in a Neubauer chamber.

### Mammalian cell culture conditions

The A549 human lung epithelial cell line (ATCC CCL-185) was used in this study. The cultures were grown and maintained in RPMI-1640 (Sigma Aldrich) supplemented with 10% heat-inactivated fetal bovine serum (JR Scientific Inc, USA) without antibiotics. The cells were grown at 37°C with 5% CO_2_ and seeded every three days when confluency neared 85%.

### Interaction of *C*. *neoformans* with host cells

2.5 × 10^5^ A549 cells were seeded in 6-well plates and grown for 22 hours. *C*. *neoformans* suspensions were prepared in RPMI-1640 to generate a ratio of 10 and 100 yeasts per host cell, representing MOI 10 and MOI100 respectively. The interactions between *C*. *neoformans* and the host cells occurred at 37°C at a 5% CO_2_ atmosphere for 6, 12 and 18 hours. The supernatant was harvested, spiked with DL-Alanine-2, 3, 3, 3-d_4_ and stored at -80°C. The experiment was performed using 4 biological replicates and 3 technical replicates.

### Sample preparation

All samples were subjected to freeze-drying prior to derivatisation. 80 μl of methoxyamine hydrochloride in pyridine (20 mg/ml) was added to each sample and incubated at 30°C with shaking for 90 minutes. The samples were added with 160 μl of MSTFA and allowed to incubate at 37°C with shaking for 30 minutes. The samples were transferred to autosampler vials and allowed to acclimatise to room temperature for 2 hours prior to the GC-MS run.

### Metabolic profiling

#### GC-MS analysis

The chromatographic analysis was performed using Agilent 7890A gas chromatographer coupled with an Agilent 5975C mass spectrometer operated at 70eV. The column used for the analysis was HP-5MS (Agilent), 30m × 250μm inner diameter and 0.25μm film thickness. The MS was operated in scan mode (start after 5 min, mass range 40–650 amu).

#### Separation and analysis of the derivatives

The oven temperature was initially held at 80°C for 2 minutes. The temperature was raised with a gradient of 5°C/min to 280°C and held for 5 minutes. The flow through the column was held constant at 1 ml He/min. Sample volumes of 1μl was injected splitless. The temperature of the inlet was 260°C, the interface temperature was 280°C and the ion source was adjusted to 230°C. The GC column was equilibrated for 6 minutes prior to each analysis. The metabolites were identified by comparison to the NIST 2005 database (version 2.0, FairCom Co., USA).

#### Multivariate data analysis

The data were normalised and arranged in a three-dimensional matrix consisting of arbitrary peak index, sample names and normalised peak area. The resulting three-dimensional data table was entered into the SIMCA (version 13.0.3, Umetrics, Sweden) software package for multivariate statistical analysis. The general clustering and trends depicting metabolite differences were analysed using unsupervised principal component analysis (PCA). Next, supervised partial least squares discriminant analysis (PLS-DA) model was generated to identify significantly altered metabolites between the different groups. The R^2^X and Q^2^Y values are good indicators of the quality of the model. The R^2^X value indicates the goodness of fit of the model based on variance while the Q^2^Y value represents the cumulative variation in Y. For these parameters, values approaching 1.0 indicates a strong predictive reliability based on the fact that the model is stable. The variable importance in the projection (VIP) values was used to identify the most significantly different metabolites associated with the infection with low and high MOI respectively. The metabolites with VIP scores of 1 and greater were selected as the discriminating metabolites between the different time points. The three-dimensional data were further validated by transformation by the fourth root and compiled into a Bray Curtis similarity matrix using PRIMER v.6 (Primer-e, Ivybridge, UK). A Spearman correlation of > 0.5 was used as an arbitrary limit to display potential correlation between the metabolite abundance and the collection time points relative to the canonical axes. The variables that were significantly discriminated between groups for both the software were considered potential biomarkers.

#### Construction of metabolic pathways

The construction and pathway analysis of potential disease progression markers was performed using MetaboAnalyst 2.0. The program is based on several databases, including KEGG (http://www.genome.jp/kegg/) and the Human Metabolome Database (http://www.hmdb.ca/) and identifies the pathways that are most significantly perturbed.

#### Effects of metabolites on growth

The *C*. *neoformans* H99 cells were grown overnight at 37°C with shaking at 240 rpm and were harvested by centrifugation at 3,000 rpm for 5 minutes. The cells were washed with PBS twice, resuspended in Sabouraud Dextrose broth (SDB) and then enumerated using a Neubauer chamber. A cell concentration of 1 × 10^5^ cells/ml was prepared in 10 ml of SDB and spiked with 16 μg/ml and 128 μg/ml of the respective discriminant metabolites. The use of 16 μg/ml and 128 μg/ml represented a moderate and high dose of the metabolites respectively. The suspension was incubated at 37°C with shaking at 240 rpm. At four-hour intervals, the OD_600nm_ was measured. The measurements were taken until the yeast reached the stationary phase. The experiment was performed in triplicates.

#### Effects of metabolites on biofilm formation

The *C*. *neoformans* H99 cells were grown overnight at 37°C with shaking at 240 rpm and were harvested by centrifugation at 3,000 rpm for 5 minutes. The cells were washed with PBS twice, resuspended in SDB and then enumerated using a Neubauer chamber. A cell concentration of 1 × 10^5^ cells/ml was seeded in 96-well plates and spiked with 16 μg/ml and 128 μg/ml of the respective discriminant metabolites. The plates were incubated at 37°C for 24 hours. The supernatant was then removed and the wells were washed with PBS twice. 100 μl of fresh SDB was added into the wells together with 10 μl of alamar blue reagent. The plates were incubated for 6 hours and visualized at an absorbance of 490 nm with a reference wavelength of 595 nm. The experiment was performed in triplicates. Amphotericin B was used as the positive control.

#### Effects of metabolites on mature biofilm

The *C*. *neoformans* H99 cells were grown overnight at 37°C with shaking at 240 rpm and were harvested by centrifugation at 3,000 rpm for 5 minutes. The cells were washed with PBS twice, resuspended in SDB and then enumerated using a Neubauer chamber. A cell concentration of 1 × 10^5^ cells/ml was seeded in 96-well plates and was incubated at 37°C for 48 hours. The supernatant was then removed and the wells were washed with PBS twice. The respective discriminant metabolites were added into the wells at concentrations of 16 μg/ml and 128 μg/ml. The plates were then incubated for 37°C for 24 hours. The supernatant was then removed and the wells were washed with PBS twice. 100 μl of fresh SDB was added into the wells together with 10 μl of alamar blue reagent. The plates were incubated for 6 hours and visualized at an absorbance of 490 nm with a reference wavelength of 595 nm. The experiment was performed in triplicates. Amphotericin B was used as the positive control.

#### Statistical analysis

Analyses of the growth and biofilm data were performed using Statistical Package for the Social Sciences (SPSS) version 20. A P value of ≤ 0.05 was considered significant.

## Results

### Non-targeted metabolite profiling of *C*. *neoformans* infection of lung epithelial cells using GC-MS

The current study focused on the determination of important metabolites secreted during infection of lung epithelial cells by *C*. *neoformans*. Using the described GC-MS conditions, metabolic footprinting of the infection of the lung epithelial cells with *C*. *neoformans* was performed. Generally, ion chromatograms do not provide any evidence of difference and similarity of complex sample sets and the results require a more sophisticated method of data analysis.

**Fig 1:**
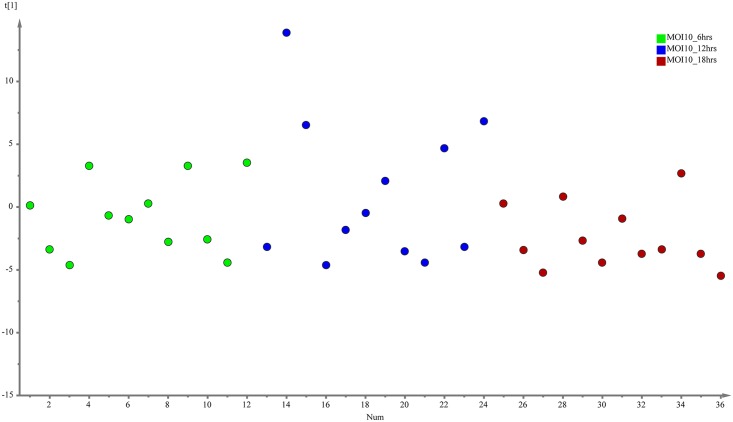
PCA model results for the metabolites secreted during infection of MOI10. Data show difference between 36 samples (4 biological replicates, 3 technical replicates of samples co-incubated for 6, 12 and 18 hours)

**Fig 2:**
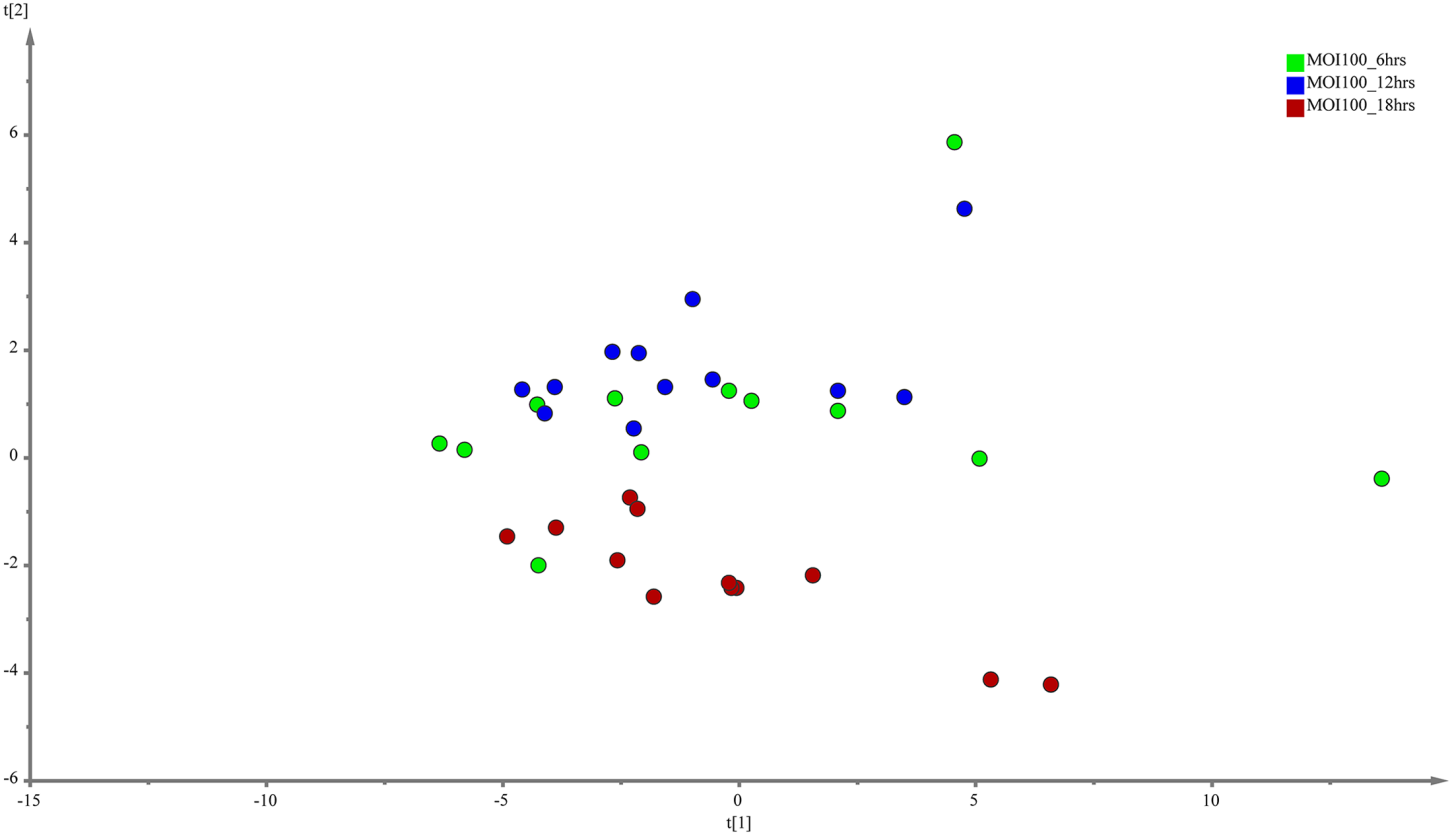
PCA model results for the metabolites secreted during infection of MOI100. Data show difference between 36 samples (4 biological replicates, 3 technical replicates of samples co-incubated for 6, 12 and 18 hours)

The evaluation of the capability of the GC-MS-based metabolomics approach to obtain the disease progression markers for early infection of *C*. *neoformans* with lung epithelial cells was performed using multivariate data analysis (PCA and PLS-DA). According to the PCA score plots, the differences of metabolite composition between the three different time points for both MOI10 and MOI100 were explained by R^2^ scores of 0.609 and 0.743 respectively (Figs 1 and 2). The results suggest that the metabolite profiles are consistent within the biological replicates and that there is a difference in the metabolic characteristics as the co-incubation state progresses.

### Discriminative metabolites for early infection stage at MOI10 and MOI100

**Fig 3:**
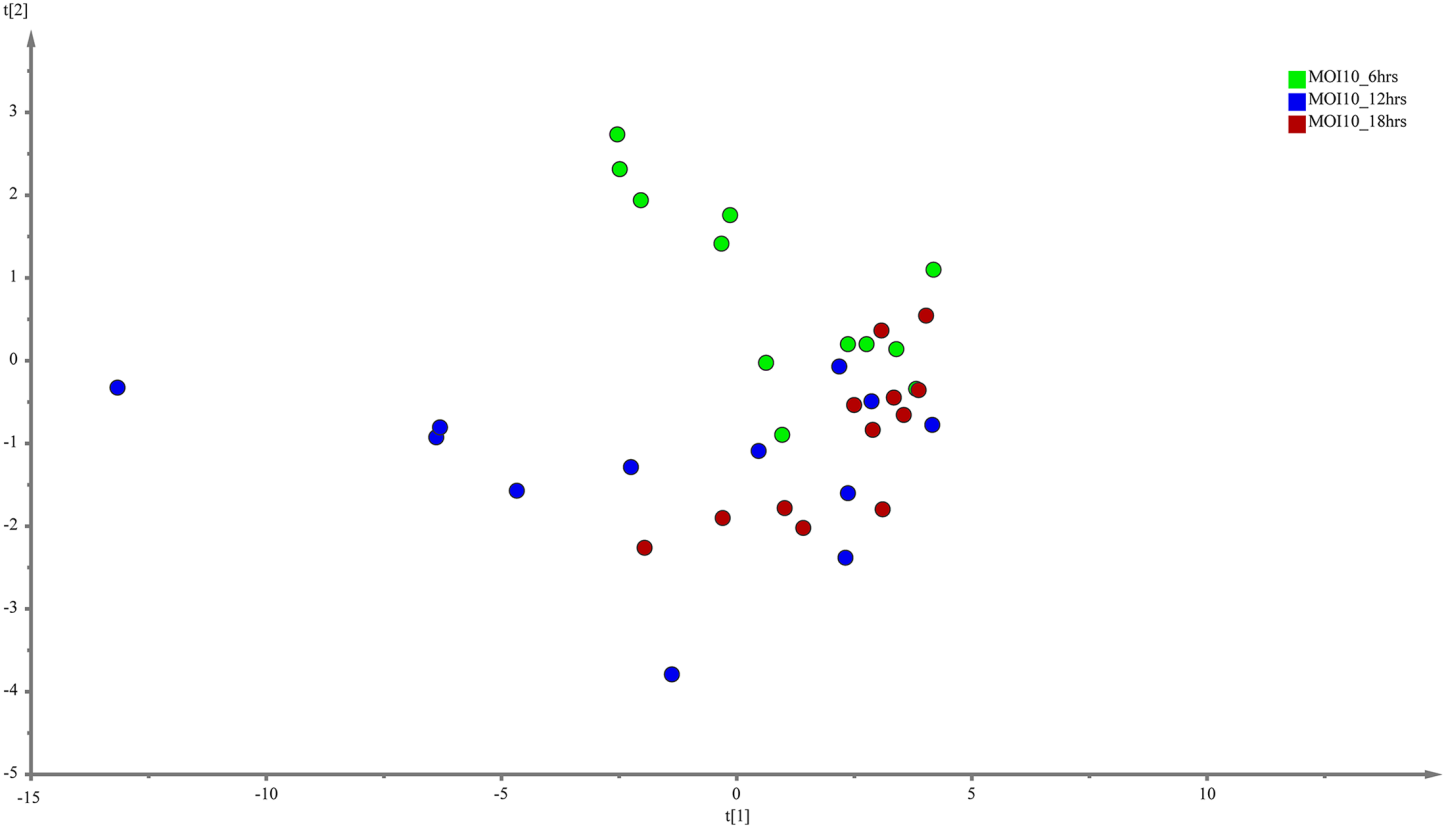
PLS-DA model results for the metabolites secreted during infection of MOI10. Data show difference between 36 samples, but the Q^2^Y values are too low for PLS-DA to be used as the sole mode of identification of discriminant metabolites.

**Fig 4:**
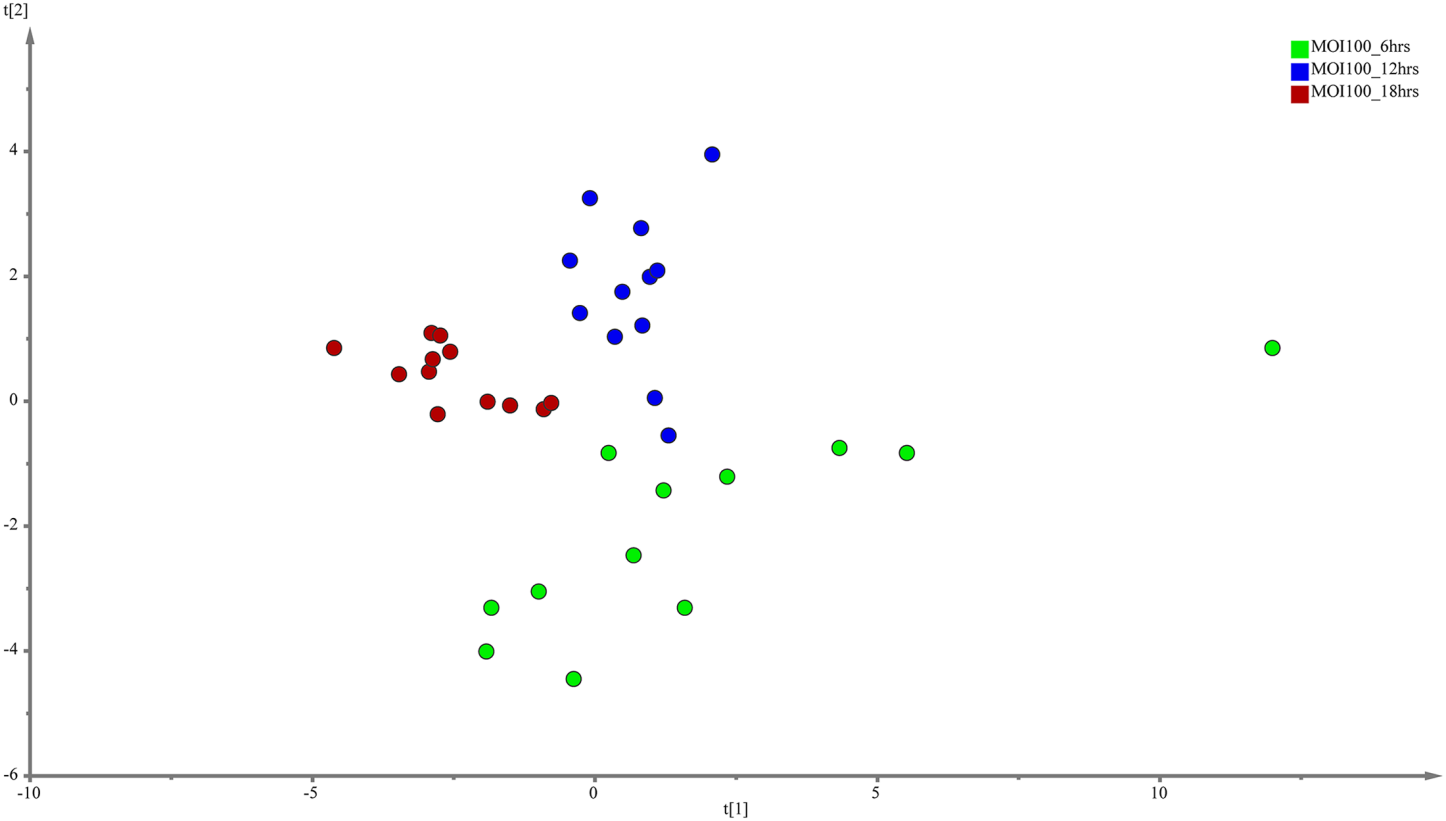
PLS-DA model results for the metabolites secreted during infection of MOI100. Data show difference between 36 samples, but the Q^2^Y values are too low for PLS-DA to be used as the sole mode of identification of discriminant metabolites.

PLS-DA was performed to search for discriminative metabolites that contributed to the separation observed between the metabolite profiles. The analysis revealed a more differential metabolic signature as time progresses for both the MOI10 and MOI100 group. PLS-DA is a commonly used method to detect hidden variables that focuses on class separation [13]. The discriminant metabolites were selected using VIP values > 1. A total of 15 metabolites were identified from the MOI10 samples, with a R^2^X value of 0.743 and a Q^2^Y score of 0.0913 while a total of 16 metabolites were identified from the MOI100 samples, with a R^2^X value of 0.63 and a Q^2^Y score of 0.306 (Figs 3 and 4).

**Fig 5:**
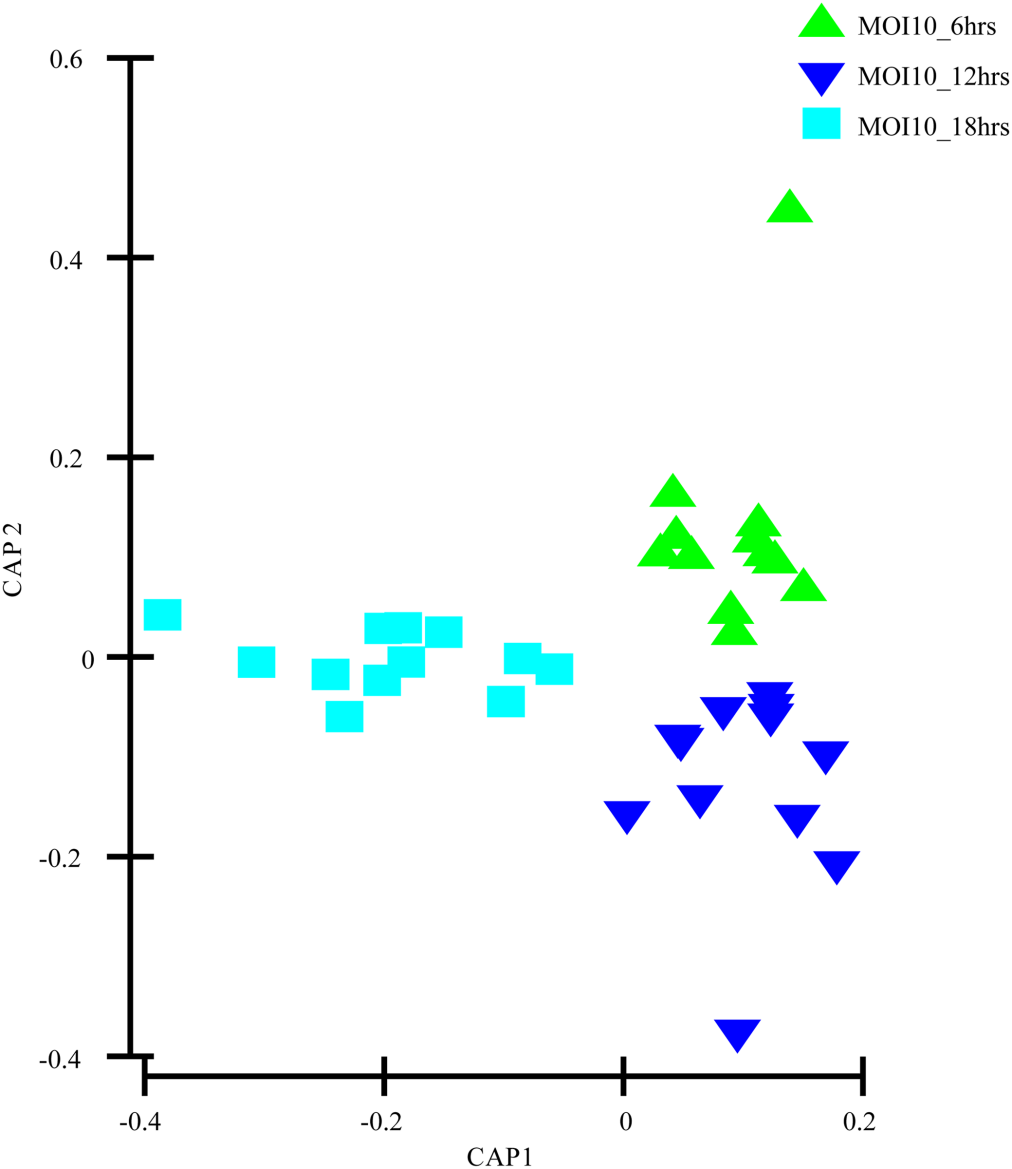
CAP model detailing the metabolites secreted during infection of MOI10. There was a distinct spread between the metabolites of the different time points. This allowed for the grouping of specific metabolites based on the time points.

**Fig 6:**
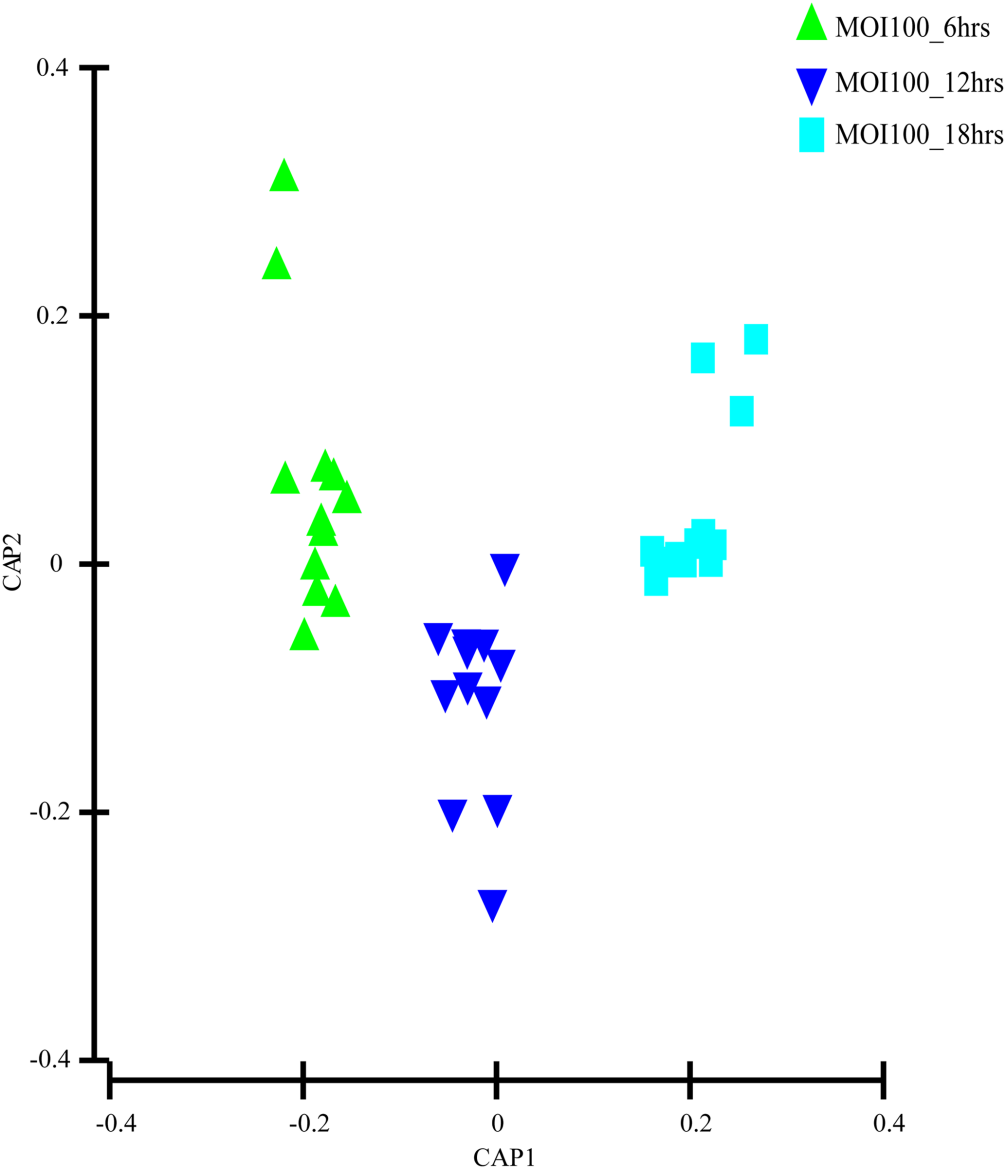
CAP model detailing the metabolites secreted during infection of MOI100. There was a distinct spread between the metabolites of the different time points. This allowed for the grouping of specific metabolites based on the time points.

As the Q^2^Y scores from the PLS-DA for both the low and high infection loads were relatively low, the predictability of the models was low and required further verification before the discriminant metabolites could be picked. Therefore, the data was subjected to transformation and Bray Curtis similarity matrix was performed and visualised using canonical analysis of principal coordinates (CAP) in PRIMER v.6. From the CAP analysis, there was a clear separation between the metabolites of the three different time points (Figs 5 and 6). The reliability of the model for both MOI10 and MOI100 were very significant, with permanova P value of less than 0.001. Based on the reliability score of both models, it was ascertained that the discriminant metabolites obtained in this analysis is reliable and could be used for further analysis. Therefore, metabolites with VIP scores more than 1.0 from the SIMCA analysis, metabolites discovered in the CAP analysis and those with P values less than 0.05 (threshold) were selected as discriminating metabolites between the three different time points. The discriminative metabolites are displayed in Table 1.

**Table 1:** The discriminative metabolites identified in the different co-incubation conditions for MOI10 and MOI100.

Co-incubation conditions	Discriminative metabolites
**MOI10_6 hours**	L-cysteine
**MOI10_12 hours and 18 hours**	Lactic acid, pantothenic acid, fumaric acid, L-tyrosine, D-fructose
**MOI100_6 hours**	L-cysteine
**MOI100_12 hours and 18 hours**	Adonitol, DL-3-phenyllactic acid, malic acid, 3-hydroxyisovaleric acid

### Metabolic pathway analyses

**Fig 7:**
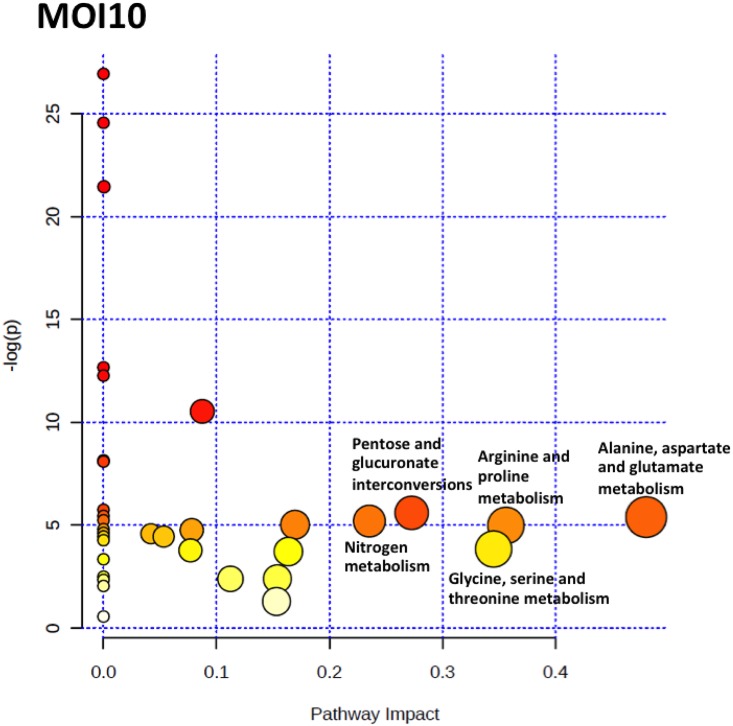
The pathway impact analysis of MOI10 samples using MetaboAnalyst 2.0. Metabolic pathways with values > 0.1 were considered to be perturbed. There were a total of 10 pathways being affected in the MOI10 samples.

**Fig 8:**
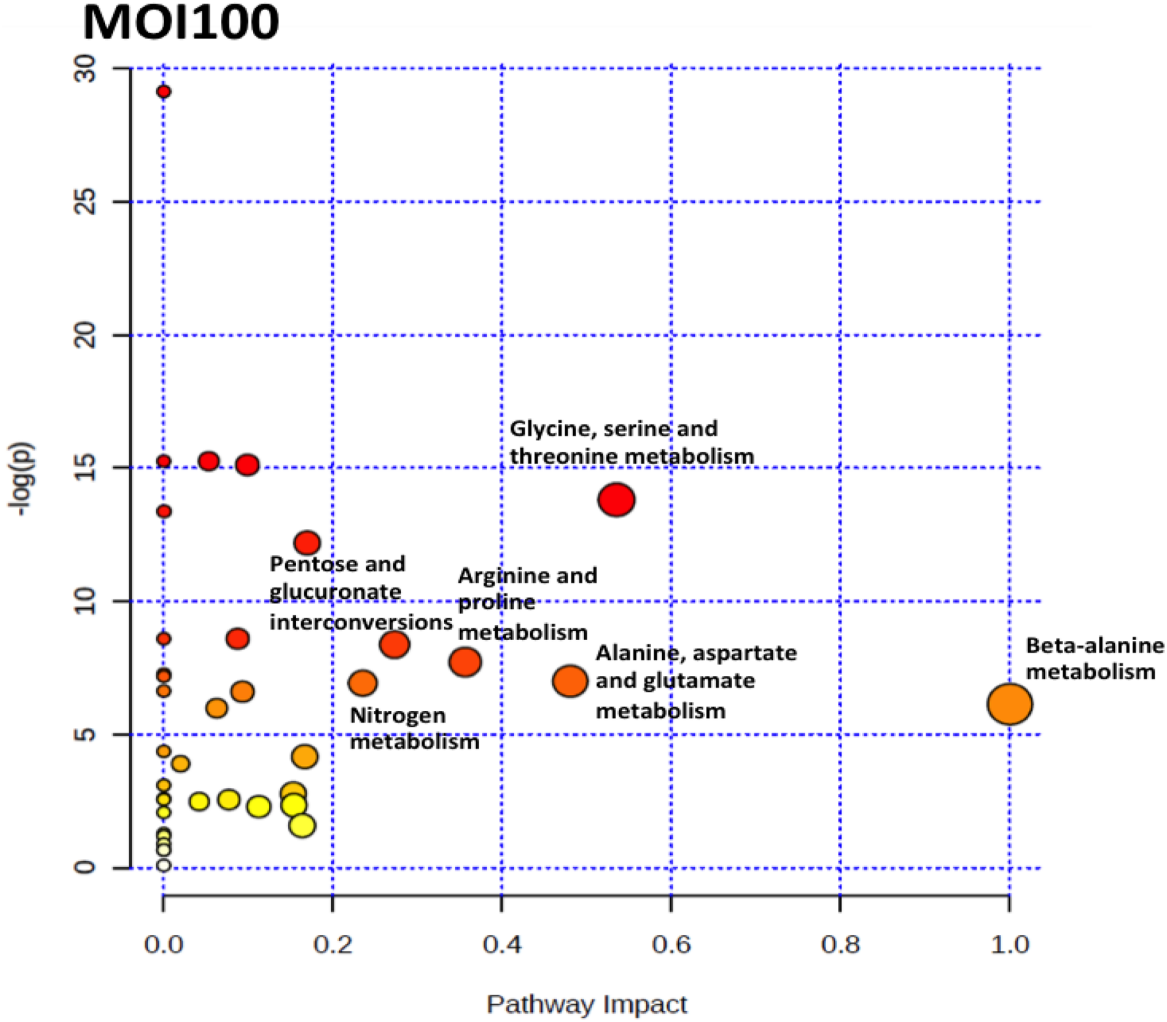
The pathway impact analysis of MOI100 samples using MetaboAnalyst 2.0. Metabolic pathways with values > 0.1 were considered to be perturbed. There were a total of 13 pathways being affected in the MOI100 samples.

To identify the pathways that are perturbed during the co-incubation of *C*. *neoformans* with the lung epithelial cells, the metabolite profile of each incubation point were analysed using MetaboAnalyst 2.0 software. MetaboAnalyst 2.0 is a web-based software that derives its predictive ability from the KEGG metabolic pathways database. The software utilizes pathway enrichment and topology analysis to identify pathways that are most significantly perturbed under the specific experimental conditions. In our analysis, it was identified that there were several pathways, including the alanine, aspartate and glutamate metabolism, nitrogen metabolism, glutathione metabolism, arginine and proline metabolism, glycine, serine and threonine metabolism, inositol phosphate metabolism, citrate cycle (TCA cycle), glyoxylate and dicarboxylate metabolism, pantothenate and CoA biosynthesis and pentose and glucuronate interconversions that were perturbed for the MOI10 samples. Similar pathways were also perturbed in the MOI100 samples but the incubation at higher MOI also resulted in the perturbance of the cysteine and methionine metabolism, beta-alanine metabolism and methane metabolism. The summary of the pathway analysis is shown in Figs 7 and 8 while the results of the pathway analysis are shown in Tables 2 and 3.

**Table 2:** Results of the pathway analysis of the MOI10 samples.

Pathways involved	Total	Hits	Raw p	-log(p)	Holm adjust	FDR	Impact
Alanine, aspartate and glutamate metabolism	20	5	0.0045453	5.3937	0.10454	0.012237	0.48039
Arginine and proline metabolism	37	6	0.0068372	4.9854	0.13175	0.014077	0.3562
Glycine, serine and threonine metabolism	26	5	0.021491	3.8401	0.2364	0.030088	0.34522
Pentose and glucuronate interconversions	12	2	0.0036959	5.6005	0.092398	0.01176	0.27273
Nitrogen metabolism	8	4	0.0055277	5.198	0.1172	0.012898	0.23529
Glutathione metabolism	23	4	0.0065873	5.0226	0.13175	0.014077	0.16949
Inositol phosphate metabolism	19	1	0.024284	3.7179	0.2364	0.031479	0.16364
Citrate cycle (TCA cycle)	20	3	0.091274	2.3939	0.57559	0.10346	0.15387
Pantothenate and CoA biosynthesis	16	2	0.27465	1.2923	0.57559	0.28272	0.15306
Glyoxylate and dicarboxylate metabolism	14	2	0.091636	2.3899	0.57559	0.10346	0.11232
Starch and sucrose metabolism	18	2	2.66E-05	10.534	0.00079823	0.00015521	0.08733
Cysteine and methionine metabolism	33	2	0.008563	4.7603	0.14713	0.015774	0.07804
Tryptophan metabolism	27	1	0.022915	3.776	0.2364	0.030847	0.07692
Sulfur metabolism	13	1	0.011704	4.4478	0.16386	0.017811	0.05319
Valine, leucine and isoleucine biosynthesis	24	4	0.010222	4.5833	0.15653	0.017036	0.04189
Glycolysis or Gluconeogenesis	24	1	4.79E-10	21.458	1.58E-08	5.59E-09	0.00039

**Table 3:** Results of the pathway analysis of the MOI100 samples.

Pathways involved	Total	Hits	Raw p	-log(p)	Holm adjust	FDR	Impact
β-Alanine metabolism	7	3	0.002181	6.128	0.045801	0.0046043	1
Glycine, serine and threonine metabolism	26	6	1.01E-06	13.803	3.44E-05	7.70E-06	0.53508
Alanine, aspartate and glutamate metabolism	20	5	0.00091393	6.9978	0.022848	0.0024807	0.48039
Arginine and proline metabolism	37	6	0.00044247	7.7231	0.012389	0.0015285	0.3562
Pentose and glucuronate interconversions	12	1	0.00022975	8.3785	0.0066629	0.00087307	0.27273
Nitrogen metabolism	8	4	0.00098542	6.9224	0.02365	0.0024964	0.23529
Glutathione metabolism	23	4	5.12E-06	12.183	0.00016369	2.78E-05	0.16949
Methane metabolism	11	2	0.015614	4.1596	0.28105	0.028254	0.16667
Inositol phosphate metabolism	19	1	0.20637	1.5781	1	0.24507	0.16364
Citrate cycle (TCA cycle)	20	3	0.096068	2.3427	1	0.12588	0.15387
Pantothenate and CoA biosynthesis	16	3	0.062474	2.773	0.93711	0.098917	0.15306
Glyoxylate and dicarboxylate metabolism	14	2	0.10137	2.289	1	0.1284	0.11232
Cysteine and methionine metabolism	33	3	2.74E-07	15.11	9.59E-06	2.60E-06	0.0988
Aminoacyl-tRNA biosynthesis	67	14	0.0013627	6.5983	0.03056	0.003046	0.09302
Starch and sucrose metabolism	18	2	0.00018486	8.5959	0.0057307	0.00078196	0.08733
Tryptophan metabolism	27	1	0.078019	2.5508	1	0.1098	0.07692
Pyrimidine metabolism	35	1	0.0025276	5.9805	0.050553	0.0050553	0.06267
Sulfur metabolism	13	1	2.38E-07	15.252	8.80E-06	2.60E-06	0.05319
Valine, leucine and isoleucine biosynthesis	24	4	0.083941	2.4776	1	0.11392	0.04189
Phenylalanine, tyrosine and tryptophan biosynthesis	22	3	0.020328	3.8958	0.34558	0.035112	0.0199
Glycolysis or Gluconeogenesis	24	1	1.56E-06	13.37	5.15E-05	9.89E-06	0.00039

### Effects of discriminant metabolites on *C*. *neoformans*

**Fig 9:**
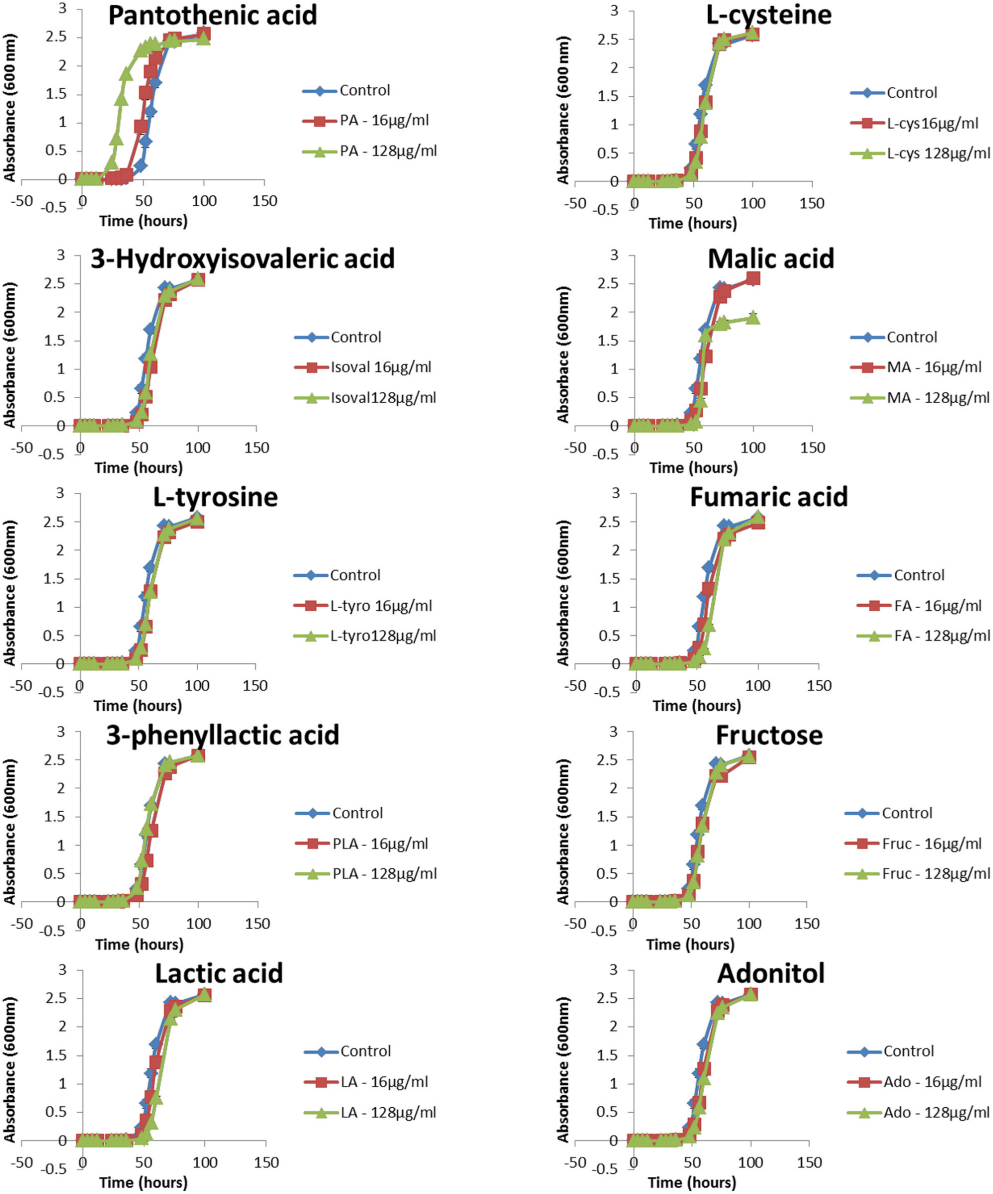
Growth curve of *C*. *neoformans* H99 after the addition of discriminant metabolites. There was a significant increase (P ≤ 0.01) in growth after addition of pantothenic acid (16 μg/ml and 128 μg/ml). * represents P ≤ 0.05; ** represents P ≤ 0.01

**Fig 10:**
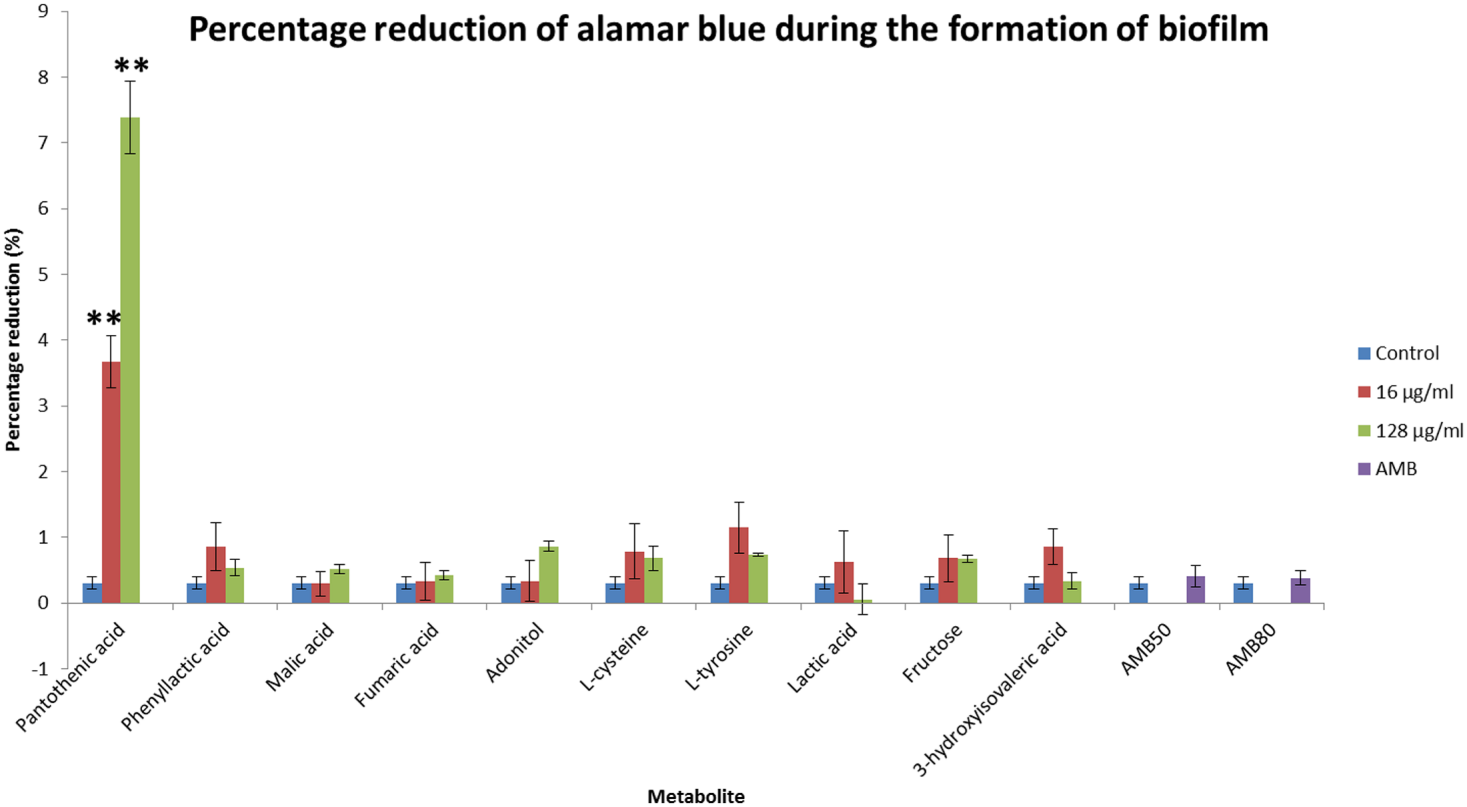
Percentage reduction in alamar blue by the discriminant metabolites during the formation of biofilm. The higher the percentage reduction, the more viable cells present; hence representing increased biofilm formation. Administration of 16 μg/ml and 128 μg/ml pantothenic acid significantly increased (P ≤ 0.01) biofilm formation. * represents P ≤ 0.05; ** represents P ≤ 0.01

**Fig 11:**
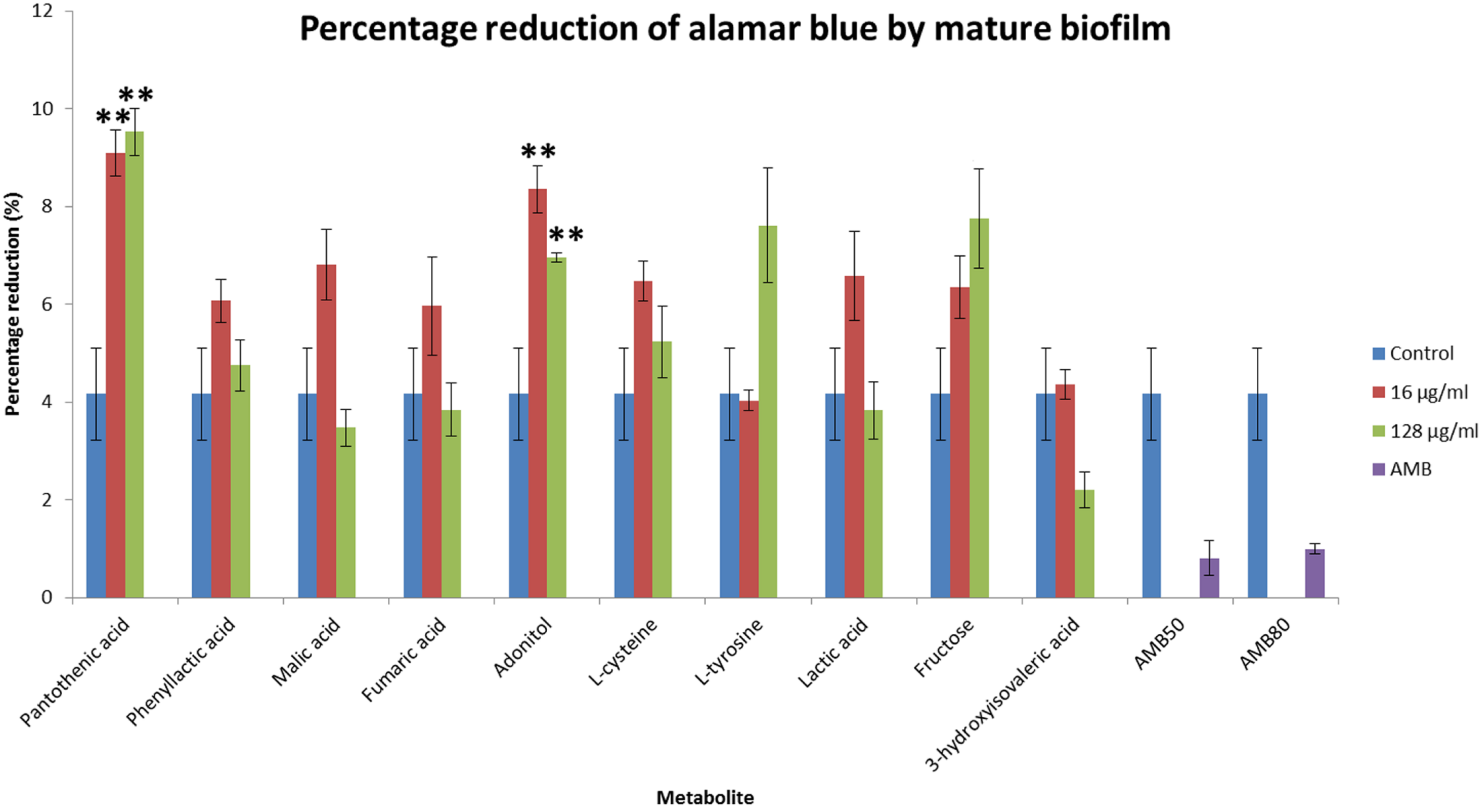
Percentage reduction in alamar blue by the discriminant metabolites on mature biofilm. The higher the percentage reduction, the more viable cells present; hence representing increased biofilm formation. Administration of 16 μg/ml and 128 μg/ml pantothenic acid and adonitol significantly increased (P ≤ 0.01) the viability of cells in the mature biofilm. * represents P ≤ 0.05; ** represents P ≤ 0.01

The effects of the discriminant metabolites on the growth and biofilm formation of *C*. *neoformans* were examined. The ability to grow at 37°C is a virulence factor of *C*. *neoformans* and the addition of pantothenic acid increased the growth rate significantly (Fig 9). The addition of pantothenic acid also caused an increase in formation of biofilm (Fig 10) while adonitol, fructose and pantothenic acid caused an increase in formation of mature biofilm (Fig 11).

## Discussion

*C*. *neoformans* is an opportunistic pathogen and inhalation by immunocompetent individuals will lead to none or relatively mild signs and symptoms. However, individuals who are immunosuppressed or immunocompromised might suffer from pulmonary infections and subsequently meningoencephalitis [2]. The burden of the disease is global but the sub-Saharan African as well as the South and South-east Asian region is the most highly affected due to the high prevalence of AIDS cases [14]. As cryptococcosis is not a notifiable disease in many countries situated in those regions, the reporting of cases will be underestimated [5, 15]. Moreover, the lack of initial symptoms also makes early detection and diagnosis difficult [5]. The emerging metabolomics field of research is able to provide a powerful platform for discovering potential novel biomarkers and biochemical pathways that can potentially distinguish healthy patients from those that are infected [16]. Changes in the expression levels of proteins have large effects on the concentrations of intermediary metabolites; therefore the metabolome is more sensitive to perturbations than either the transcriptome or proteome [17]. Metabolic footprinting has been the preferred choice over metabolic fingerprinting due to its high-throughput analysis capabilities. Metabolic footprinting also does not involve complex procedures, is less time consuming, more reliable and reproducible when compared to intracellular metabolite analysis [6, 17]. Ultimately, it would allow non-invasive screening of patient samples and offer an earlier chance of disease diagnosis.

In this study, the co-incubation of *C*. *neoformans* with lung epithelial cells was performed to simulate cryptococcal infection in the primary site of infection, the lungs. The use of A549 cells in the study was to mimic the alveolar cells that *C*. *neoformans* would interact with upon deposition into the lung as the pulmonary epithelial cells represent the first line of defence against external pathogens [18] At the moment, there are no primary cell lines to mimic similar cells in humans. Besides, cancerous cell lines have also been used as the host to examine the molecular mechanisms behind the infection of *Shigella flexneri* [19]. The lung infection would lead to metabolites being secreted into the surrounding microenvironment, therefore the culture supernatant from the co-incubation studies with different initial infection loads were analysed using GC-MS coupled with multivariate data analysis. We identified a difference in the PCA score for the three time points in both the MOI10 and MOI100 samples. However, the PLS-DA analysis could not clearly distinguish between the different time points in each infection load. Therefore, a canonical analysis of principal coordinates (CAP) was performed to further validate the data. The results from the CAP analysis revealed that the discriminative metabolites from the MOI 10 samples were 97.2% accurate while those of the MOI100 samples were 100% accurate. Based on the pathway analysis obtained in this study, it was found that there were 10 pathways perturbed in the MOI10 infection ratio while 12 pathways were perturbed in the MOI100 infection ratio. The pathways that were perturbed in both the MOI samples were similar with the MOI100 infection pathway having additional perturbance in β-alanine metabolism and methane metabolism.

In both the MOI10 and MOI100 samples, the majority of the pathways perturbed were from the central carbon metabolism and biosynthesis of amino acids. Due to the presence of two different sets of cells in the co-incubation condition, the glucose in the media would be used up quickly. This was also seen during the early infection stages in the lung where *C*. *neoformans* has to adapt to microenvironments where glucose is limited [7, 20]. The ICL1 gene encodes isocitrate lyase, an important enzyme in the glyoxylate cycle. In conditions of low glucose during pulmonary infection, the expression of ICL1 would be elevated and cause an overall enhancement of the glyoxylate and dicarboxylate cycle [7, 21]. The MLS1 gene for malate synthase, another enzyme in the glyoxylate and dicarboxylate cycle, was also found to be upregulated in pulmonary infections. The glyoxylate and dicarboxylate cycle plays an important role in maintaining infection but not for the virulence of *C*. *neoformans* [7, 20]. During nutrient starvation, *Candida albicans* would also undergo a switch from glycolysis to gluconeogenesis to elicit a starvation response via the glyoxylate cycle. This adaptation mechanism contributes to the host colonization and pathogenesis by *C*. *albicans* [22]. During early infection stages, the enzymes in the TCA cycle are also affected. The enzymes aconitase and succinate dehydrogenase are normally elevated during pulmonary infection to enable *C*. *neoformans* to utilize six-carbon sugars and respiration for energy production [7, 23].

In microenvironments with limited glucose such as the early infection stages of the lung, there would be a need for alternative carbon sources such as acetate, lactate, fatty acids or amino acids for the pathogen to proliferate [20]. During pulmonary infection, the metabolic response of *C*. *neoformans* causes the production of glucose and acetyl-CoA through the mobilization of glycogen [7]. The production of acetyl-CoA from pyruvate and acetate during pulmonary infections was caused by the upregulation of genes for acetyl-coenzyme A synthetase (ACS1), pyruvate decarboxylase and aldehyde dehydrogenase [7]. The production of acetyl-CoA by *C*. *neoformans* during infection is crucial for the synthesis of chitin in the cell wall and the O-acetylation of the capsule [7]. The ATP-citrate lyase (*acl1*) plays an important role in metabolic adaptation as ACL1 transcript levels are elevated upon encountering macrophages [24]. The reduction in the production of acetyl-CoA due to the deletion of ACL1 in *acl1* mutants alters glucan levels in the cell wall, resulting in defective capsule attachment and shedding [24]. Depletion of acetyl-CoA stores would also lead to acetylation of the capsule and this would cause defects in the capsule structure as well as altered antibody binding, complement activation and tissue accumulation [25, 26]. This defect contributed to the growth defects of *acl1* mutants of *C*. *neoformans* and also resulted in complete avirulence in a mouse model [24, 25, 26]. The genes from the three main pathways for producing cytosolic acetyl-CoA were upregulated during the interaction of *C*. *neoformans* during infection [27]. The three pathways are the β-oxidation of fatty acids by the enzyme *Mfe2*, from acetate through acetate synthetase (*Acs1*) and from citrate through the actions of *acl1*. Deletion mutants of the ACL1 gene completely attenuated virulence while the *mfe2* and *acs1* mutants exhibited reduced virulence [7, 27]. This proves that acetyl-CoA is vital for maintenance of cryptococcal infection in mammalian hosts.

Of particular interest in this study is the perturbance in the β-alanine metabolism observed exclusively in the MOI100_18 hour sample. Previously, it was indicated that yeast had to acquire pantothenic acid and β-alanine exogenously for growth. A research group discovered that *Saccharomyces cerevisiae* is capable of *de novo* synthesis of β-alanine and pantothenic acid from methionine via the S-adenosylmethionine and the polyamine pathway [28]. During early infection stages of soybeans by *Rhizoctonia solani*, there was an increase in the production of β-alanine. The increase in β-alanine was important to increase the biosynthesis of coenzyme A (CoA), which is crucial in conferring resistance to biotic stress [29]. During interaction in oxygen-limiting microenvironments, the transcription factor Sre1 in *C*. *neoformans* will increase the expression of genes involved in the pantothenate and coenzyme-A metabolism. This will in turn increase the production of acetyl-CoA, the initial substrate for ergosterol biosynthesis [30]. Pantothenic acid has been reported to be secreted by *C*. *neoformans* in conditions of low glucose and was observed in the media during the plateau phase of growth [31].

In concordance with our earlier findings, pantothenic acid caused a significant increase in *C*. *neoformans* growth rate even at concentrations of 16 μg/ml. Pantothenic acid has been reported to increase growth of *C*. *neoformans* in a dose-dependent manner [31]. Pantothenic acid and even its precursor, β-alanine, have been reported to be able to increase growth of *Saccharomyces cerevisiae* [32]. Pantothenic acid is taken into the cell by pantothenate transporters localised to the plasma membrane known as Fen2p while the uptake of β-alanine was primarily done by the yeast’s general amino acid, permease known as Gap1p [32].

Pantothenic acid also caused an increase in biofilm formation and mature biofilms in *C*. *neoformans* while adonitol caused an increase in the formation of mature biofilms. Pantothenic acid has been reported to increase biofilm formation but the mechanism remains unknown [31]. The formation of biofilm in *Lactobacillus rhamnosus luxS* mutants was enhanced by the supplementation of pantothenic acid. Although the exact pathway remains unknown, but it was postulated that the mutants require a more complex nutritional requirement for proliferation. Biofilm growth is enhanced under starvation conditions as it often induces the production of an extracellular matrix [33]. Fungi living closely with lichens are able to utilize adonitol as a food source and convert it to mannitol [34]. Growth of *C*. *neoformans* with fructose also increases the production of mannitol. Increase in mannitol would lead to an increase in the size of the capsule and thus increase formation of biofilm through the shedding and accumulation of large amounts of GXM into the biofilm extracellular matrix [35, 36]. The increase in the adhesion of *C*. *neoformans* in the lungs would lead to colonization and the formation of biofilm, ultimately causing chronic lung infection [37]. The secretion of capsular polysaccharide promotes biofilm formation and may act as a fungal reservoir, shielding the planktonic cells from the host immune response [37].

The increase in the secretion of both pantothenic acid and β-alanine was reflected in the perturbation of the β-alanine metabolism and pantothenate and CoA biosynthesis during the co-incubation. The mechanisms that are underlying this perturbation and the components involved in this process require further study. The targeting of the pantothenate and CoA biosynthesis pathway as a potential target in antimicrobial studies has been explored but it has not been confirmed as an option for *C*. *neoformans* infection [32]. This study provides a new link between the metabolic disturbances seen during pulmonary infection by *C*. *neoformans* under immunocompromised conditions. The metabolites found, particularly pantothenic acid, may potentially be an important metabolite to distinguish between individuals with *C*. *neoformans* infection and possibility of providing a new means of diagnosis. However, the limitation of the study remains the inability to determine the cells responsible for the metabolites secreted into the culture supernatant due to the closeness in similarity between the sizes of both cells. Nevertheless, a study identified the metabolic response of dengue infection on endothelial cells without differentiating the metabolites secreted by the pathogen or the host and only focusing on the host cells [38]. In this study, due to the larger volume of *C*. *neoformans* cells compared to the A549 cells, the metabolites obtained in this study were most likely secreted by the fungal cells. Human cells are also unable to secrete several metabolites that were discovered in this study such as pantothenic acid and β-alanine [28]. A study on *Shigella flexneri* infection of human epithelial cells by Kentner and colleagues in 2014 also postulated that the metabolic response of the infection would be dominated by metabolites secreted by the host cells rather than the pathogen as a larger volume of host cells were used [19]. An alternative for future studies would be to ^13^C-label the *C*. *neoformans* cells and the resulting interaction can be analysed using mass-spectrometry-based chromatographic methods to differentiate the metabolites produced by the pathogen and the host cells [39].

## Conclusion

Our results have shown that there were 10 discriminative metabolites discovered from the infection of *C*. *neoformans* with lung epithelial cells at an MOI of 10 and 100. L-cysteine and pantothenic acid were the only two discriminant metabolites that were present in both the infection loads. The infection at both infection loads led to a perturbance in the glyoxylate and dicarboxylate cycle, indicating the metabolic adaptation displayed by *C*. *neoformans* during infection of mammalian cells. The presence of pantothenic acid increases the growth rate of *C*. *neoformans* as well as in biofilm conditions while adonitol increased the viability of the biofilm cells. Since there was a perturbance observed in the β-alanine metabolism pathway in the MOI100 samples, its contribution to *C*. *neoformans* during infection is unclear and warrants further investigation. The use of animal models for infection could be done in the future to determine the metabolite profile associated with high and low infection loads in an *in vivo* environment.

## Supporting Information

S1 Dataset Raw normalized mass spectral data used for multivariate analysis. (XLSX)

## References

[1] Liu OW, Chun CD, Chow ED, Chen C, Madhani HD, Noble SM. Systemic genetic analysis of virulence in the human fungal pathogen *Cryptococcus neoformans*. Cell. 2008; 135: 174–188. 10.1016/j.cell.2008.07.046 18854164 PMC2628477

[2] Li SS, Mody CH. Cryptococcus. Proc Am Thorac Soc. 2010; 7: 186–196. 10.1513/pats.200907-063AL 20463247

[3] Cogliati M. Global molecular epidemiology of *Cryptococcus neoformans* and *Cryptococcus gattii*: an atlas of the molecular types. Scientifica. 2013; 2013 10.1155/2013/675213 PMC3820360 24278784

[4] Choo KK, Chong PP, Ho ASH, Yong PVC. Effect of inoculum size and culture age on the cellular properties and host-pathogen interactions of *Cryptococcus neoformans*. Br Microbiol Res J. 2015; 7(2): 100–108.

[5] Tay ST, Rohani MY, Soo Hoo TS, Hamimah H. Epidemiology of cryptococcosis in Malaysia. Mycoses. 2009; 53: 509–514. 10.1111/j.1439-0507.2009.01750.x 19627508

[6] Mapelli V, Olsson L, Nielsen J. Metabolic footprinting in microbiology: methods and applications in functional genomics and biotechnology. Cell. 2008; 26(9): 490–497. 10.1016/j.tibtech.2008.05.008 18675480

[7] Hu G, Cheng P, Sham A, Perfect JR, Kronstad JW. Metabolic adaptation in *Cryptococcus neoformans* during early murine pulmonary infection. Mol Microbiol. 2008; 69(6): 1456–1475. 10.1111/j.1365-2958.2008.06374.x 18673460 PMC2730461

[8] Choi JN, Kim J, Kim J, Jung WH, Lee CH. Influence of iron regulation on the metabolome of *Cryptococcus neoformans*. PLoS ONE. 2012; 7(7). 10.1371/journal.pone.0041654 PMC3402442 22911836

[9] Bai Y, Zhang H, Sun X, Sun C, Ren L. Biomarker identification and pathway analysis by serum metabolomics of childhood lymphoblastic leukemia. Clin Chim Acta. 2014; 436: 207–216. 10.1016/j.cca.2014.05.022 24909874

[10] Bogdanov M, Matson WR, Wang L, Matson T, Saunders-Pullman R, Bressman SS, et al Metabolic profiling to develop blood biomarkers for Parkinson’s disease. Brain. 2008; 131: 389–396. 10.1093/brain/awm304 18222993

[11] Saha DC, Xess I, Jain N. Evaluation of conventional & serological methods for rapid diagnosis of cryptococcosis. Indian J Med Res. 2008; 127: 483–488. 18653913

[12] Arora S, McDonald RA, Toews GB, Huffnagle GB. Effect of a CD4-depleting antibody on the development of Cryptococcus neoformans-induced allergic bronchopulmonary mycosis in mice. Infect Immun. 2006; 74(7): 4339–4348. 16790808 10.1128/IAI.01989-05 PMC1489708

[13] Kouskoumvekaki I, Panagiotou G. Navigating the human metabolome for biomarker identification and design of pharmaceutical molecules. J Biomed Biotechnol. 2011; 2011 10.1155/2011/525497 PMC2948926 20936122

[14] Park BJ, Wannemuehler KA, Marston BJ, Govender N, Pappas PG, Chiller TM. Estimation of the current global burden of cryptococcal meningitis among persons living with HIV/AIDS. AIDS. 2009; 23(4): 525–530. 10.1097/QAD.0b013e328322ffac 19182676

[15] Liew KL, Jee JM, Yong VC. *In silico* approaches in the identification of *Cryptococcus neoformans* chemoreceptors. Afr J Biotechnol. 2012; 11(46): 10469–10472.

[16] Zhang A, Sun H, Yan G, Han Y, Ye Y, Wang X. Urinary metabolic profiling identifies a key role for glycocholic acid in human liver cancer by ultra-performance liquid-chromatography coupled with high-definition mass spectrometry. Clin Chim Acta. 2013; 418: 86–90. 10.1016/j.cca.2012.12.024 23313056

[17] Kell DB, Brown M, Davey HM, Dunn WB, Spasic I, Oliver SG. Metabolic footprinting and systems biology: the medium is the message. Nat Rev Microbiol. 2005 10.1038/nrmicro1177 15953932

[18] David J, Bell RE, Clark GC. Mechanisms of disease: Host-pathogen interactions between Burkholderia species and lung epithelial cells. Front Cell Infect Microbiol. 2015 10.3389/fcimb.2015.00080 PMC4649042 26636042

[19] Kentner D, Martano G, Callon M, Chiquet P, Brodmann M, Burton O, et al *Shigella* reroutes host cell central metabolism to obtain high-flux nutrient supply for vigorous intracellular growth. PNAS. 2014; 111(27):9929–9934. 10.1073/pnas.1406694111 24958876 PMC4103312

[20] Kronstad J, Saikia S, Nielson ED, Kretschmer M, Jung W, Hu G, et al Adaptation of *Cryptococcus neoformans* to mammalian hosts: integrated regulation of metabolism and virulence. Eukaryot Cell. 2012; 11(2): 109–118. 10.1128/EC.05273-11 22140231 PMC3272904

[21] Rude TH, Toffaletti DL, Cox GM, Perfect JR. Relationship of the glyoxylate pathway to the pathogenesis of *Cryptococcus neoformans*. Infect Immunn. 2002; 70(10): 5684–5694. 10.1128/IAI.70.10.5684-5694.2002 PMC128360 12228298

[22] Mayer FL, Wilson D, Hube B. Candida albicans pathogenicity mechanisms. Virulence. 2013; 4(2): 119–128. 10.4161/viru.22913 23302789 PMC3654610

[23] Flores C, Rodriguez C, Petit T, Gancedo C. Carbohydrate and energy-yielding metabolism in non-conventional yeasts. FEMS Microbiol Rev. 2000; 24: 507–529. 10978549 10.1111/j.1574-6976.2000.tb00553.x

[24] Griffiths EJ, Hu G, Fries B, Caza M, Wang J, Gsponer J et al A defect in ATP-citrate lyase links acetyl-CoA production, virulence factor elaboration and virulence in *Cryptococcus neoformans*. Mol Microbiol. 2012; 86(6): 1404–1423. 10.1111/mmi.12065 23078142 PMC3524413

[25] Janbon G, Himmelreich U, Moyrand F, Improvisi L, Dromer F. Cas1p is a membrane protein necessary for the O-acetylation of the *Cryptococcus neoformans* capsular polysaccharide. Mol Microbiol. 2001; 42: 453–467. 11703667 10.1046/j.1365-2958.2001.02651.x

[26] Kozel TR, Levitz SM, Dromer F, Gates MA, Thorkildson P, Janbon G. Antigenic and biological characteristics of mutant strains of *Cryptococcus neoformans* lacking capsular O acetylation or xylosyl side chains. Infect Immun. 2003; 71: 2868–2875. 12704160 10.1128/IAI.71.5.2868-2875.2003 PMC153297

[27] Kretschmer M, Wang J, Kronstad JW. Peroxisomal and mitochondrial beta-oxidation influence the virulence of the pathogenic fungus *Cryptococcus neoformans*. Eukaryot Cell. 2012; 11: 1042–1054. 10.1128/EC.00128-12 22707485 PMC3416069

[28] White WH, Gunyuzlu PL, Toyn JH. *Saccharomyces cerevisiae* is capable of de novo pantothenic acid biosynthesis involving a novel pathway of β-alanine production from spermine. J Biol Chem. 2001; 276: 10794–10800. 11154694 10.1074/jbc.M009804200

[29] Aliferis KA, Faubert D, Jabaji S. A metabolic profiling strategy for the dissection of plant defense against fungal pathogens. PLoS ONE. 2014; 9(11). 10.1371/journal.pone.0111930 PMC4219818 25369450

[30] Bien CM, Chang YC, Nes WD, Kwon-Chung KJ, Espenshade PJ. *Cryptococcus neoformans* Site-2 protease is required for virulence and survival in the presence of azole drugs. Mol Microbiol. 2009; 74(3): 672–690. 10.1111/j.1365-2958.2009.06895.x 19818023 PMC2917040

[31] Albuquerque P, Nicola AM, Nieves E, Paes HC, Williamson PR, Silva-Pereira I, et al Quorum sensing-mediated, cell density-dependent regulation of growth and virulence in *Cryptococcus neoformans*. MBio. 2014; 5(1). 10.1128/mBio.00986-13 PMC3884061 24381301

[32] Spry C, Kirk K, Saliba KJ. Coenzyme A biosynthesis: an antimicrobial drug target. FEMS Microbiol Rev. 2008; 32: 56–106. 10.1111/j.1574-6976.2007.00093.x 18173393

[33] Lebeer S, De Keersmaecker SCJ, Verhoeven TLA, Fadda AA, Marchal K, Vanderleyden J. Functional analysis of *luxS* in the probiotic strain *Lactobacillus rhamnosus* GG reveals a central metabolic role important for growth and biofilm formation. J Bacteriol. 2007; 189(3): 860–871. 17098890 10.1128/JB.01394-06 PMC1797292

[34] Gostincar C, Muggia L, Grube M. Polyextremotolerant black fungi: oligotrophism, adaptive potential and a link to lichen symbioses. Front Microbiol. 2012; 3 10.3389/fmicb.2012.00390 PMC3492852 23162543

[35] Guimarães AJ, Frases S, Cordero RJB, Nimrichter L, Casadevall A, Nosanchuk JD. *Cryptococcus neoformans* responds to mannitol by increasing capsule size *in vitro* and *in vivo*. Cell Microbiol. 2010; 12(6): 740–753. 10.1111/j.1462-5822.2010.01430.x 20070311 PMC2891871

[36] Robertson EJ, Casadevall A. Antibody-mediated immobilization of *Cryptococcus neoformans* promotes biofilm formation. Appl Environ Microbiol. 2009; 75(8): 2528–2533. 10.1128/AEM.02846-08 19251903 PMC2675221

[37] Patel D, Desai GM, Frases S, Cordero RJB, DeLeon-Rodriguez CM, Eugenin EA, et al Methamphetamine enhances *Cryptococcus neoformans* pulmonary infection and dissemination to the brain. MBio. 2013; 4(4). 10.1128/mBio.00400-13 PMC3735193 23900172

[38] Birungi G, Chen SM, Loy BP, Ng ML, Li SFY. Metabolomics approach for investigation of effects of dengue virus infection using EA.hy926 cell line. J Proteome Res. 2010; 9: 6523–6534. 10.1021/pr100727m 20954703

[39] Beste DJV, Nöh K, Niedenführ S, Mendum TA, Hawkins ND, Ward JL, et al ^13^C-flux spectral analysis of host-pathogen metabolism reveals a mixed diet for intracellular *Mycobacterium tuberculosis*. Chem Biol. 2013; 20(8): 1012–1021. 10.1016/j.chembiol.2013.06.012 23911587 PMC3752972

